# p*K*_a_ Calculations of GPCRs: Understanding Protonation States in Receptor Activation

**DOI:** 10.1021/acs.jcim.4c01125

**Published:** 2024-08-16

**Authors:** Carlos A. V. Barreto, João N. M. Vitorino, Pedro B.P.S. Reis, Miguel Machuqueiro, Irina S. Moreira

**Affiliations:** † PhD Programme in Experimental Biology and Biomedicine, Institute for Interdisciplinary Research (IIIUC), University of Coimbra, Casa Costa Alemão, 3030-789 Coimbra, Portugal; ‡ CNC—Center for Neuroscience and Cell Biology, Center for Innovative Biomedicine and Biotechnology, University of Coimbra, 3004-504 Coimbra, Portugal; § BioSI—Instituto de Biossistemas e Ciências Integrativas, Faculdade de Ciências, Universidade de Lisboa, 1749-016 Lisboa, Portugal; ∥ Department of Life Sciences, University of Coimbra, Calçada Martim de Freitas, 3000-456 Coimbra, Portugal

## Abstract

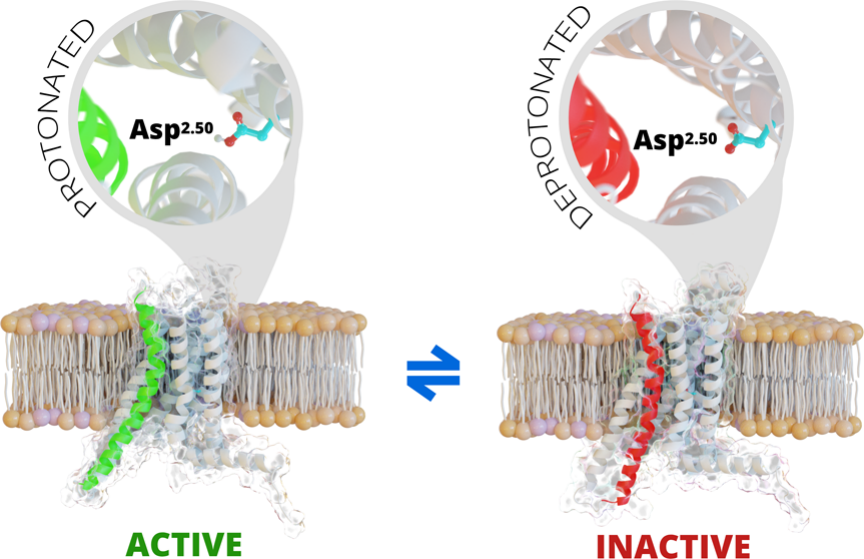

The increase in the available G protein-coupled receptor (GPCR) structures has been pivotal in helping to understand their activation process. However, the role of protonation–conformation coupling in GPCR activation still needs to be clarified. We studied the protonation behavior of the highly conserved Asp^2.50^ residue in five different class A GPCRs (active and inactive conformations) using a linear response approximation (LRA) p*K*_a_ calculation protocol. We observed consistent differences (1.3 p*K* units) for the macroscopic p*K*_a_ values between the inactive and active states of the A2AR and B2AR receptors, indicating the protonation of Asp^2.50^ during GPCR activation. This process seems to be specific and not conserved, as no differences were observed in the p*K*_a_ values of the remaining receptors (CB1R, NT1R, and GHSR).

## Introduction

G protein-coupled receptors (GPCRs) are the largest family of membrane proteins in the human body and are the target of one-third of the drugs approved in the market. This superfamily is crucial for communication between intracellular and extracellular environments. GPCRs change their conformation during receptor activation to accommodate intracellular partners (G protein or arrestin). The rapid increase in resolved GPCR structures has enabled large-scale comparisons between states, which showed a common activation process for GPCRs, particularly for class A.^1,2^

**Figure 1:**
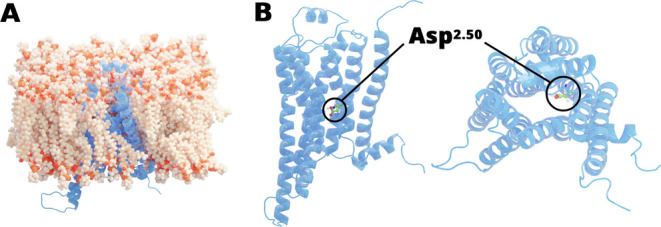
A2AR structure embedded in a lipid bilayer (A) and side and top views of the Asp^2.50^ residue (B). The protein is represented by a light blue cartoon, the lipids are shown as orange spheres, and the key Asp residue is shown as green sticks.

Protonation of the conserved residues at the receptor’s core has been suggested as a key event during GPCR activation.^3−7^ Although some protonation candidates have been studied, the most studied and probable candidate is Asp^2.50^ (superscripts represent the Ballesteros–Weinstein numbering^8^) (Figure 1).

This residue is conserved in 94% of class A GPCRs and is critical for signaling.^9^ Furthermore, mutations of Asp^2.50^ to an uncharged residue resulted in the decrease or absence of G protein signaling.^10,11^ However, the exact protonation state of Asp^2.50^ remains unclear. Vanni et al.^12^ and Ranganathan et al.^13^ have published studies on the protonation of Asp^2.50^ in β-adrenergic receptors. Vanni et al.^12^ studied interactions at the core of the β_1_ adrenergic receptor (B1AR) and β_2_ adrenergic receptor (B2AR) with protonated and deprotonated versions of Asp^2.50^ in an inactive state. They found a strong correlation between the presence of an ionic lock and protonated Asp^2.50^, suggesting that protonation of this residue stabilizes the inactive state.^12^ Ranganathan et al.^13^ performed molecular dynamics (MD) free-energy calculations on B2AR inactive and active crystal structures and reported a p*K*_a_ shift of 3.2–3.7 units upon activation, suggesting a protonated Asp^2.50^ in the active conformation. However, it is unclear whether this variation occurred within the pH range.

Poisson–Boltzmann (PB)-based p*K*_a_ calculations rely on single conformations and assume that they are representative of the macroscopic p*K*_a_ values. This is not often the case, as many structures are biased toward a preferred protonation state, thus propagating that propensity toward unphysical p*K*_a_ predictions. On the other hand, the use of a linear response approximation (LRA) method, while simple, has been successful at predicting p*K*_a_ values for proteins by averaging over the p*K*_half_ (p*K*_a_ values in single conformations) in the protonated and deprotonated states.^14−16^ This approach is much simpler than other more advanced (and computationally expensive) methods, such as constant-pH MD (CpHMD), while often yielding similar results.^17^

Here, we applied our p*K*_a_ calculation protocol to membrane proteins^18^ on five different class A GPCRs with inactive and active resolved structures, simulated with protonated and deprotonated Asp^2.50^ residues. The LRA calculations provide p*K*_a_ estimations of this conserved aspartate and help to clarify the role of (de)protonation in the earliest stages of (de)activation of these receptors.

## Methods

### Molecular Mechanics/Molecular Dynamics

#### Protein Structures and System Building

The active and inactive structures of five different GPCRs were selected: adenosine A_2A_ receptor (A2AR), B2AR, cannabinoid receptor 1 (CB1R), ghrelin receptor (GHSR), and neurotensin receptor type 1 (NT1R) (Table S1 of the Supporting Information). The fully refined structures of each receptor were retrieved from GPCRdb,^19,20^ and additional molecules (ligands, ions, water) present in the PDB file were deleted. The orientation of the membrane was retrieved from the orientations of proteins in the membrane (OPM) database.^21^

Two systems were built for active and inactive conformations: one with Asp^2.50^ deprotonated and the other with Asp^2.50^ protonated. All other titratable residues remained in their most abundant protonation state at pH 7.0. CHARMM-GUI^22−24^ was used to insert the receptors into a bilayer lipid membrane in a cubic simulation box hydrated with TIP3P and 0.15 M NaCl. The N- and C-termini were capped with ACE/CT1 groups. A binary lipid bilayer was built around the receptor structure with 1-palmitoyl-2-oleoyl-*sn*-*glycero*-3-phosphocholine (POPC) and cholesterol (CHL1) (ratio 9:1), with 100 lipids per leaflet. A total of 2000 water molecules were added to the system (100 water molecules per lipid).

#### Molecular Dynamics Simulation Parameters

MD simulations were performed using GROMACS 2018.4^25^ and CHARMM36m force field.^26^ The systems were simulated by using the NPT ensemble. A V-rescale thermostat was used with a coupling constant of 0.1 ps to achieve and maintain the desired temperature (310 K). Pressure coupling was performed using a semi-isotropic Parrinello–Rahman barostat at 1 bar with a compressibility of 4.5 × 10^–5^ bar^–1^ and a coupling constant of 1.0 ps. Electrostatic interactions were computed with the particle-mesh Ewald (PME) method,^27^ with a Fourier grid of 0.12 nm and a cutoff of 1.2 nm for direct contributions. Lennard-Jones interactions were computed using a nonbonded neighbor pair list with a cutoff of 1.2 nm, using the Verlet scheme. Solute bonds were constrained using a Parallel LINear Constraint Solver (p-LINCS).^28^ The steepest descent algorithm was used to minimize the initial energy of the system through a 50,000-step run. The systems were then initialized for 21 ns: a first step of 1 ns with an NVT ensemble with force constraints of 1000 kJ nm^–2^ mol^–1^ on the protein backbone and side chain and lipid’s heavy atoms; and four runs of 5 ns with an NPT ensemble each with successively lower force constraints (1000; 500; 100; 10 kJ nm^–2^ mol^–1^). For each receptor, three replicas of 100 ns were performed for each scenario inactive/Asp^2.50^ deprotonated (InactDeprot), inactive/Asp^2.50^ protonated (InactProt), active/Asp^2.50^ deprotonated (ActDeprot), and active/Asp^2.50^ protonated (ActProt). Snapshots containing receptors and membranes were extracted at 100 ps intervals for the p*K*_a_ calculations.

### Poisson–Boltzmann/Monte Carlo Calculations

All p*K*_a_ calculations were performed using the PypKa tool,^29^ which is a python module for PB-based p*K*_a_ calculations using DelPhi.^30^ For each system, the MD trajectory frames were extracted and used to compute the p*K*_half_ values for the key Asp^2.50^ residue and surrounding titrable residues. The distinction between p*K*_half_ and p*K*_a_ is based on the fact that the former refers to a microscopic energy calculated from a single conformation, whereas the latter is a macroscopic free energy calculated by using an ensemble of conformations. PB calculations were carried out with dielectric constants (ϵ) of 4 and 80 assigned to the solute and solvent, respectively. The ϵ in LRA calculations has a double role since it compensates for both the lack of polarization effects and the need for a conformational reorganization introduced upon protonation changes.^17,18,31^ An ionic strength of 0.1 M was also added. The default DelPhi databases for charges and radii adapted from G54A7 were used. A pH range −10 – +20 was used in the Monte Carlo (MC) method with calculations at 0.25 pH intervals.

### Linear Response Approximation

LRA was performed to estimate the macroscopic p*K*_a_ values of Asp^2.50^. The formalism based on the two end points follows the following equation
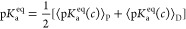
1where p*K*_a_^eq^(c) is the p*K*_a_ of the protein in conformation *c*, and the angle brackets indicate the average values of the peptide conformations sampled from the MD runs with the site protonated (P) and deprotonated (D). From the MC runs, we obtained Asp^2.50^ average protonation at all pH values. For each pH value, the average protonations of all 3003 snapshots (1001 per replicate) were again averaged and used in a Henderson–Hasselbalch formalism to calculate ensemble pH-dependent p*K*_a_ values. The resulting pH vs p*K*_a_ curves for protonated and deprotonated species of each system were averaged to produce the final LRA curve. The method’s estimation of the macroscopic p*K*_a_ of Asp^2.50^ is at the point closest to the intersection point between the averaged curve and the *y* = *x* function (when pH equals p*K*_a_). The error for this value was obtained using the Jackknife resampling approach, calculating the standard error of the mean for the p*K*_a_ values of Asp^2.50^ in the three combinations of replicates while leaving one out. Because the A2AR^ActProt^ system possesses two very different p*K*_half_ populations, only the high p*K*_half_ conformations were used for this system, and jackknife resampling was performed analogously by splitting this sampling three-way.

### Analyses

#### State Stability Analysis

Structural analyses of the systems were performed to assess the activation state of the receptors throughout the simulation. Previously described metrics, usually used to distinguish inactive and active states, have been used,^1,32,33^ such as the TM3–TM6 distance measured between Cα of 3.50 and 6.34; the TM3–TM7 distance measured between Cα of 3.50 and 7.53; the root-mean-square deviation (RMSD) of the NPxxY motif backbone relative to the inactive structure; the activation index (A^100^), described in Ibrahim et al.^33^ This index integrates five interhelical distances into one value that separates the different activation states.^33^ Values below zero were considered inactive; values between 0 and 55 were labeled as intermediate, and values above 55 were considered active conformations.

#### Activation Site Analysis

The activation sites analyzed (transmission switch, Na^+^ pocket area, hydrophobic lock area, and TM3–TM7 distance) followed the common activation pathway proposed by Zhou et al.^1^ and Hauser et al.^2^

The transmission switch shows the repacking between residues 3.40, 5.51, 6.44, and 6.48, which initiates the activation signal. Transmission switch changes were analyzed using 3.40–6.48 and 5.51–6.44 distances.

The Na^+^ pocket area looks at the binding site of the sodium ion where Asp^2.50^ resides, which collapses and initiates the movement of TM7 toward TM3. This area was calculated by defining a triangle where the sides are the distances between the selected residue side chains and using Herons’s formula to calculate the area. To determine the Na^+^ pocket, the three following distances were used: the distance between the center of mass (COM) of 2.50 side chain and a COM of 3.39 side chain; the distance between a COM of 2.50 side chain and a COM of 7.49 side chain; and the distance between a COM of 3.39 side chain and a COM of 7.49 side chain.

The hydrophobic lock is a cluster of hydrophobic residues at the core of the receptor that is essential for the stable interaction between TM3 and TM6 in the inactive state. This area was calculated in a manner similar to that used for calculating the Na^+^ pocket area. The distances used for this area were: the distance between COM of 3.43 side chain and COM of 6.40 side chain; the distance between COM of 3.43 side chain and COM of 6.41 side chain; and the distance between COM of 6.40 side chain and COM of 6.41 side chain.

Lastly, the TM3–TM7 distance, measured between 3.43 and 7.53 side chains, looks at the repacking of the NPxxY region that is essential to stabilize the TM3–TM7 interaction at the active site.

This set of metrics allowed us to investigate the activation state in more detail throughout the entire receptor structure (from the extracellular to the intracellular end). All analyses were performed using the GROMACS 2018.4 tools.^25^

## Results and Discussion

In this study, we used the active and inactive structures of five class A GPCRs to investigate changes in the protonation state of Asp^2.50^ during GPCR activation. All-atom MD simulations were conducted for each system with protonated and deprotonated Asp^2.50^.

### Activation State Stability

We used several metrics to evaluate our simulations’ equilibration and assess the stability of the initial activation state on each system. The plateaus obtained in the RMSD values of the GPCR TM region (after the initial 20 ns) indicated a good level of equilibration and convergence between replicates (Figure S1 of the Supporting Information).

To assess the activation state of GPCRs, we used the distances between TM3–TM6, TM3–TM7, and the RMSD of the NPxxY motif to the inactive state. These metrics follow the largest rearrangements of GPCR activation with the opening of the binding crevice and have been successfully used to differentiate the activation state of GPCR.^32,34−36^ The two pairwise distances clearly separated the inactive and active systems, sustained throughout the simulations with only a few exceptions. One replica of the CB1R^ActDeprot^ system showed a decrease in TM3–TM6 and an increase in TM3–TM7 (Figures S2 and S3 of the Supporting Information). RMSD of the NPxxY motif shows separation and stability throughout the simulation, although not as clear as the other metrics (Figure S4 of the Supporting Information). Nevertheless, when the RMSD values were plotted against the two interhelical distances (Figures S5 and S6 of the Supporting Information), two clear clusters were formed (one for inactive snapshots and another for active snapshots). B2AR and CB1R also showed a separation between the deprotonated and protonated active states. To further assess the activation state, the A^100^ index was calculated. This index integrates five additional interhelical distances into one value.^33^ This metric again shows distinct values for active and inactive systems that are maintained throughout the simulation time (Figure S7 of the Supporting Information). Overall, these metrics confirmed that the initial activation states were maintained throughout the MD simulations.

### A2AR^ActProt^ Shows Two Different p*K*_a_ Populations

**Figure 2:**
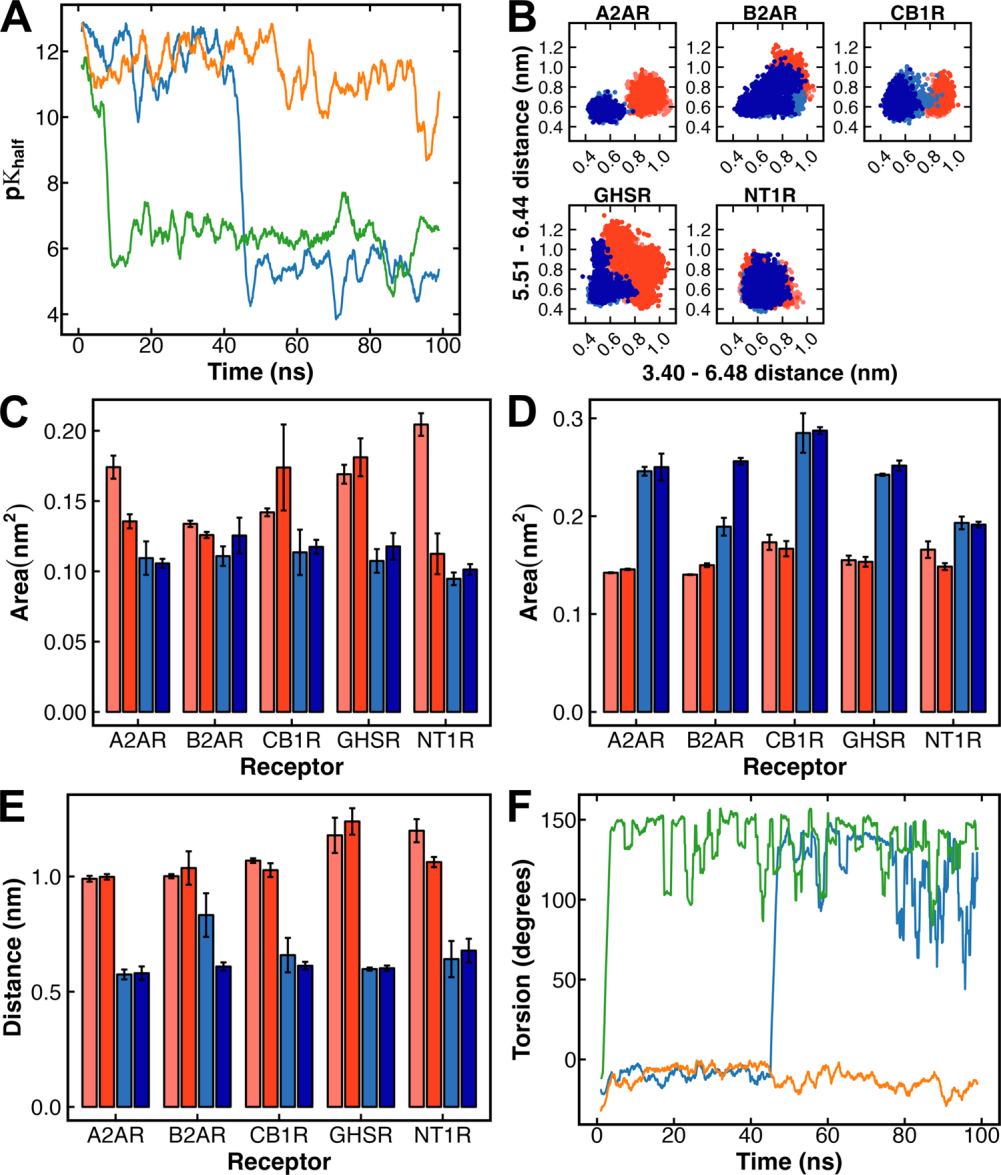
Microscopic structural analysis. (A) Time evolution of the p*K*_half_ values for the A2AR^ActProt^ system. (B–E) The GPCR activation site analysis using the transmission switch (B), the Na^+^ pocket area (C), the hydrophobic lock area (D), and the TM3 – TM7 distance (E). (F) Time evolution of the CHI2 torsion angles for the A2AR^ActProt^ system. For plots *A* and F, a floating window average (2 ns) was used for both plots, and replicas were color-coded as R1—blue, R2—orange, R3—green. For plots *B*–*E*, systems are color-coded as InactDeprot is light-red, InactProt is dark-red, ActDeprot is light blue, and ActProt is dark-blue.

The p*K*_half_ calculations of Asp^2.50^ were performed for all conformations of the studied systems (Figure S8 in the Supporting Information). Most systems also showed well-equilibrated time series. However, the activated and protonated A2AR^ActProt^ system showed a strong decrease in p*K*_half_ values, effectively resulting in two subpopulations of values (Figure 2A).

The p*K* range of the second population (5–8) is the same as that of the inactivated receptor. To understand whether this p*K*_half_ change was linked to any microscopic structural change, we investigated known GPCR activation sites, Asp^2.50^ side-chain torsion, and the H-bond network throughout the simulation time. The activation sites analyzed (transmission switch, Na^+^ pocket area, hydrophobic lock area, and TM3–TM7 distance) followed the common activation pathway proposed by Zhou et al.^1^ and Hauser et al.^2^ The transmission switch shows the repacking between residues 3.40, 5.51, 6.44, and 6.48, which initiates the activation signal, whereas the Na^+^ pocket area looks at the binding site of the sodium ion where Asp^2.50^ resides that collapses and initiates the movement of TM7 toward TM3. The hydrophobic lock is a cluster of hydrophobic residues at the core of the receptor that is essential for the stable interaction between TM3 and TM6 in the inactive state. The TM3–TM7 distance, measured between 3.43 and 7.53, looks at the repacking of the NPxxY region essential to stabilize the TM3–TM7 interaction in the active site. These metrics allowed us to investigate the activation state in more detail throughout the entire receptor structure (from the extracellular to the intracellular end). However, no link was found to the p*K*_half_ changes in A2AR^ActProt^, nor with any other system values (Figures S9–S13 of the Supporting Information). It is important to note that these metrics showed distinct values between inactive and active systems except for the transmission switch distances on NT1R, although no distinction was possible between the deprotonated and protonated systems (Figure 2B–E).

Analysis of the microscopic environment of Asp^2.50^ showed that the CHI2 torsion angle in A2AR^ActProt^ followed the same variation as the p*K*_half_ values, indicating a flip of the H-bound oxygen from Asp^2.50^ (Figures 2F and S14 in the Supporting Information). This flip occurred in two of the three replicates of the active conformation of the system, and the Asp^2.50^ H-bond network also shifted slightly with the CHI2 angle change (Figure S15 of the Supporting Information). In particular, the H-bond between Asp^2.50^ and Asn^1.50^ showed similar variations in the CHI2 torsion angles and p*K*_half_ profiles.

After the flip, the Asp^2.50^ proton was exposed to the Na^+^ pocket, suggesting a coupling between the p*K*_a_ value of this residue and the allosteric effect of Na^+^ on GPCR activation. Mutating Asp^2.50^ to uncharged residues reduced the agonist-induced signaling and in some cases Na^+^ sensitivity while maintaining ligand binding, demonstrating that this residue is crucial for the Na^+^ allosteric effect and GPCR signaling.^37,38^ It has also been proposed that the acidic character, hence deprotonation, of Asp^2.50^ is essential for Na^+^ stabilization and to prevent the collapse of this pocket, which is necessary for proper GPCR activation. Unsurprisingly, Vickery et al.^39^ have also observed a correlation between the distance of the Na^+^ ion from the binding pocket and the p*K*_a_ of Asp^2.50^ in muscarinic receptor 2, where increased distance was correlated with higher p*K*_a_ values. They also noted that the active state of the receptor was stabilized by the protonation of aspartate and tended to revert to an inactive state when the residue was charged.

### Two Receptors Showed Activation-Coupled p*K*_a_ Changes

**Figure 3:**
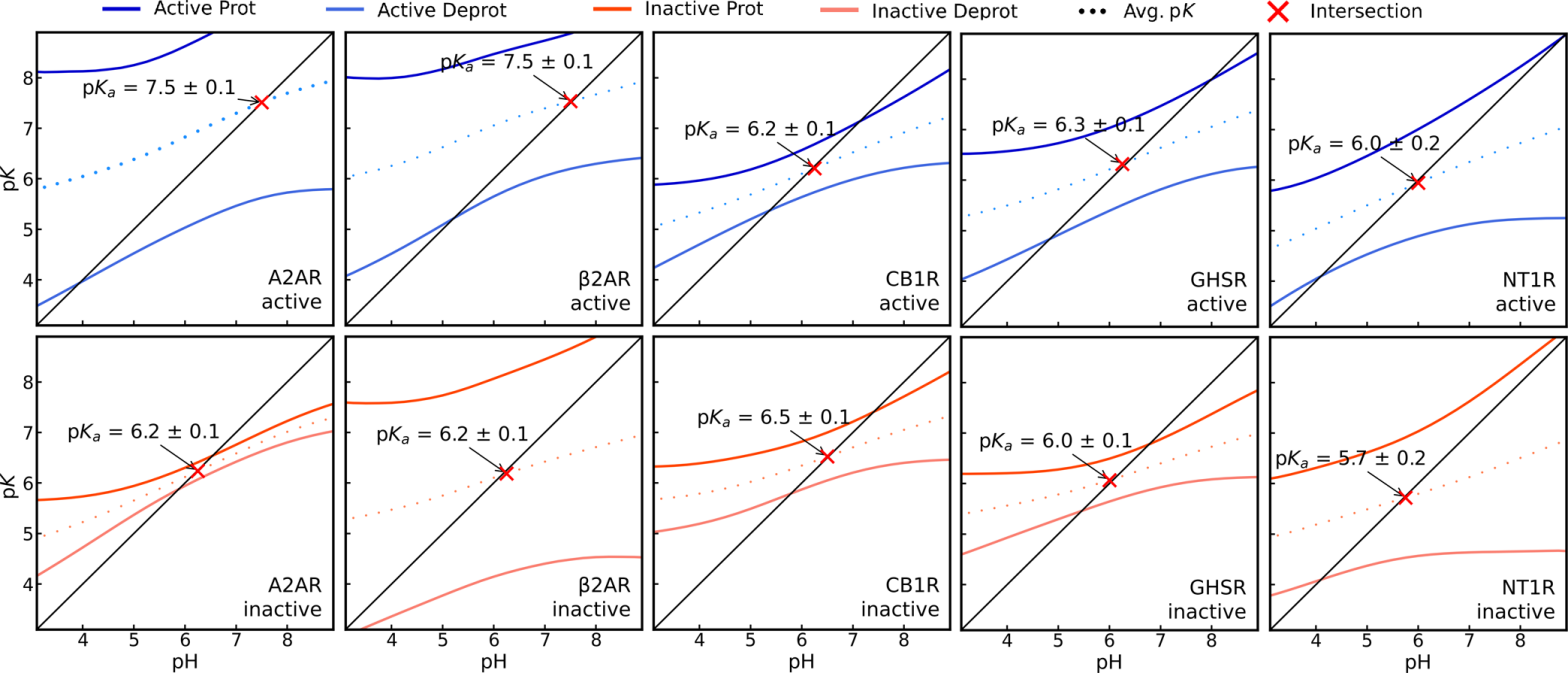
Linear response approximation plots for all studied systems. The pH vs p*K* intersection (red cross) in each graph indicates the macroscopic p*K*_a_ prediction for Asp^2.50^ in each activation state. The error values were calculated using Jackknife resampling.

The LRA method provides an estimation of the macroscopic p*K*_a_ of the activation state of each receptor. In the A2AR^ActProt^ system, two Asp^2.50^ p*K*_half_ populations exist (Figure 2A). To ensure that the receptor is fully activated in the LRA protocol, we used conformations from the ensemble with only higher p*K*_half_ values. The criteria for classifying a specific conformational ensemble as active is usually based on geometric properties that lack the high level of detail of a side-chain position. We identified and proposed Asp^2.50^ side-chain flipping as a possible microswitch to inactivation, and the calculated p*K*_half_ values are an excellent metric to help us split the conformational ensemble. The macroscopic p*K*_a_ values obtained for Asp^2.50^ showed a difference of 1.3 p*K* units between the inactive (p*K*_a_ = 6.2) and active (p*K*_a_ = 7.5) states (Figure 3).

The same values were observed for B2AR, whereas the NT1R, GHSR, and CB1R systems showed the same protonation level (Δp*K*_a_ ∼ 0.3) upon activation (Figure 3). To the best of our knowledge, this is the first study to investigate this phenomenon using multiple GPCRs. Two previous studies on Asp^2.50^ of β-adrenergic receptors showed conflicting conclusions. Vanni et al. results suggested a protonated Asp residue in the inactive state, while Ranganathan et al.’s calculations indicated protonation during activation.^12,13^ Since the active states occur in the physiological pH range, the p*K*_a_ values observed for the A2AR and B2AR systems indicate that a significant level of protonation in this residue should occur during GPCR activation. This is in agreement with the work of Ranganathan et al., who reported an increase in the p*K*_a_ units in the active structure of B2AR. In the same study, an estimate based on A2AR crystal structures also pointed to the protonation of Asp during activation.^13^

Interestingly, the number of structures of GPCRs with resolved sodium ions near Asp^2.50^ is very small and limited to the α, δ, and γ branches of class A.^38^ A2AR, B2AR, and CB1R are part of the α branch. However, only the first two receptors have structures (in the case of A2AR) or structures of closely related receptors (in the case of B2AR) with the ions resolved, which are the only receptors that show a p*K*_a_ shift between inactive and active structures. For CB1R, although no resolved structure includes a Na^+^ ion in the pocket, functional studies have suggested high Na^+^ sensitivity,^40,41^ which does not seem to agree with our results. Remarkably, structures with Na^+^ ions bound to the receptor have not been reported for the β-branch (NT1R and GHSR) GPCRs. Nevertheless, functional studies on NT1R suggest some Na^+^ sensitivity,^42−44^ whereas GHSR has been shown to bind Na^+^ and act as a negative allosteric modulator of G protein recruitment,^45^ which is in disagreement with our findings where no p*K*_a_ shift was observed between their inactive and active states.

## Conclusions

Recent advancements in structural resolution have been instrumental in delineating the differences between active and inactive states across various GPCR families.^1,2^ Nevertheless, key features such as the coupling between protonation changes and the conformational transitions of the activation process need to be clarified and included in our models. In this work, we performed p*K*_a_ calculations on MD-extracted conformations of inactive and active structures of five different GPCRs. Our LRA calculations showed consistent differences in the p*K*_a_ of the Asp^2.50^ residue between the two activation states for A2AR and B2AR, indicating protonation of this residue during GPCR activation. Furthermore, the A2AR^ActProt^ p*K*_half_ values showed a sharp decrease in the p*K*_half_ values during some MD replicates. Our analysis showed no link between this change and the GPCR activation microdomain distances except for a flip in the protonated carboxylic group of Asp^2.50^. This flip slightly shifted the H-bond network around Asp^2.50^ and positioned the proton inside the Na^+^ pocket. The coupling between the Asp residue protonation and these GPCRs’ activation is biologically significant as it provides a pH dependence for this mechanism, with consequences in specific cellular environments and tumors.

For the other three receptors (CB1R, NT1R, and GHSR), the differences in the p*K*_a_ values were insignificant. They indicated no Asp^2.50^ protonation during the activation process, contrary to the published data showing the sensitivity of these receptors to the Na^+^ ion. To address these discrepancies, it is crucial to conduct a comprehensive study of various factors, including ligand type and binding partners, modifications in known structural motifs, and the presence of Na^+^ ions, which can influence the protonation state of Asp^2.50^ during GPCR activation. We emphasize the importance of examining individual receptors using advanced computational methods, such as CpHMD, to gain clearer insights into the dynamic interplay between the protonation of this key residue and GPCR activation.

## Data Availability

The GROMACS package is freely available software for MD simulations and can be downloaded at https://manual.gromacs.org/2018.4/download.html. PypKa is a freely available software used for the MM/PB calculation and can be downloaded at https://github.com/mms-fcul/PypKa. It is also available as a web-based application at https://pypka.org/. PyMOL v2.5 is also a free software for molecular visualization. It can be downloaded from https://pymol.org/2. Finally, Blender is a free and open-source software for 3D modeling and rendering, downloadable from https://www.blender.org/.

## Supporting Information Available

Table with receptor structures used in this work; time series of several structural properties and p*K*_a_ values. Scatter plots of several structural properties and GPCR activation site analysis (PDF)
Initial and final states and topologies for each system. Example MDP file to run MD simulation. Python script to run LRA analysis (ZIP)
