# Supplementary material for: pKa Calculations of GPCRs: Understanding Protonation States in Receptor Activation
Source: J Chem Inf Model. 2024 Aug 16;64(17):6850–6. doi: 10.1021/acs.jcim.4c01125 (PMC11388449; doi:10.1021/acs.jcim.4c01125)
Supplement: Supplementary file 1 — ci4c01125_si_001.pdf [file ci4c01125_si_001.pdf]

Supporting Information:

pK_a Calculations of GPCRs: Understanding Protonation States in Receptor Activation

Carlos A.V. Barreto,[†,‡,∥] João N.M. Vitorino,[¶,∥] Pedro B.P.S. Reis,[¶] Miguel Machuqueiro,[*,¶] and Irina S. Moreira[*,‡,§]

†*PhD Programme in Experimental Biology and Biomedicine, Institute for Interdisciplinary Research (IIIUC), University of Coimbra, Casa Costa Alemão, 3030-789 Coimbra, Portugal*

‡*CNC - Center for Neuroscience and Cell Biology, Center for Innovative Biomedicine and Biotechnology, University of Coimbra, 3004-504 Coimbra, Portugal*

¶*BioISI – Instituto de Biossistemas e Ciências Integrativas, Faculdade de Ciências, Universidade de Lisboa, 1749-016, Lisboa, Portugal*

§*Department of Life Sciences, University of Coimbra, Calçada Martim de Freitas, 3000-456 Coimbra, Portugal*

∥*Equal Contribution*

E-mail: machuque@ciencias.ulisboa.pt; irina.moreira@cnc.uc.pt

Phone: +351-21-7500112; +351-23-1249170

Tables

Table S1: Receptor structures used and their main characteristics. Apart from PDB-ID 4BUO[S1], which is *Rattus norvegicus*, all structures are from the Human species.

**Receptor**	**PDB-ID**	**Activation State**	**Resolution (Å)**	**Method**	**Ligand Modality**	**Partner**
A2AR	5IU4[S2]	Inactive	1.7	X-ray	Antagonist	—
	5G53[S3]	Active	3.4	X-ray	Agonist	Gs (αs)
B2AR	2RH1[S4]	Inactive	2.4	X-ray	Inverse agonist	—
	3SN6[S5]	Active	3.2	X-ray	Agonist	Gs (αs)
CB1R	5U09[S6]	Inactive	2.6	X-ray	Inverse agonist	—
	6KPG[S7]	Active	3.0	cryo-EM	Agonist	Gi/o (αi1)
GHSR	6KO5[S8]	Inactive	3.3	X-ray	Antagonist	—
	7F9Y[S9]	Active	2.9	cryo-EM	Agonist	Gq/11 (αq)
NT1R	4BUO[S1]	Inactive	2.8	X-ray	Agonist	—
	6OS9[S10]	Active	3.0	cryo-EM	Agonist	Gi/o (αi1)

Figures

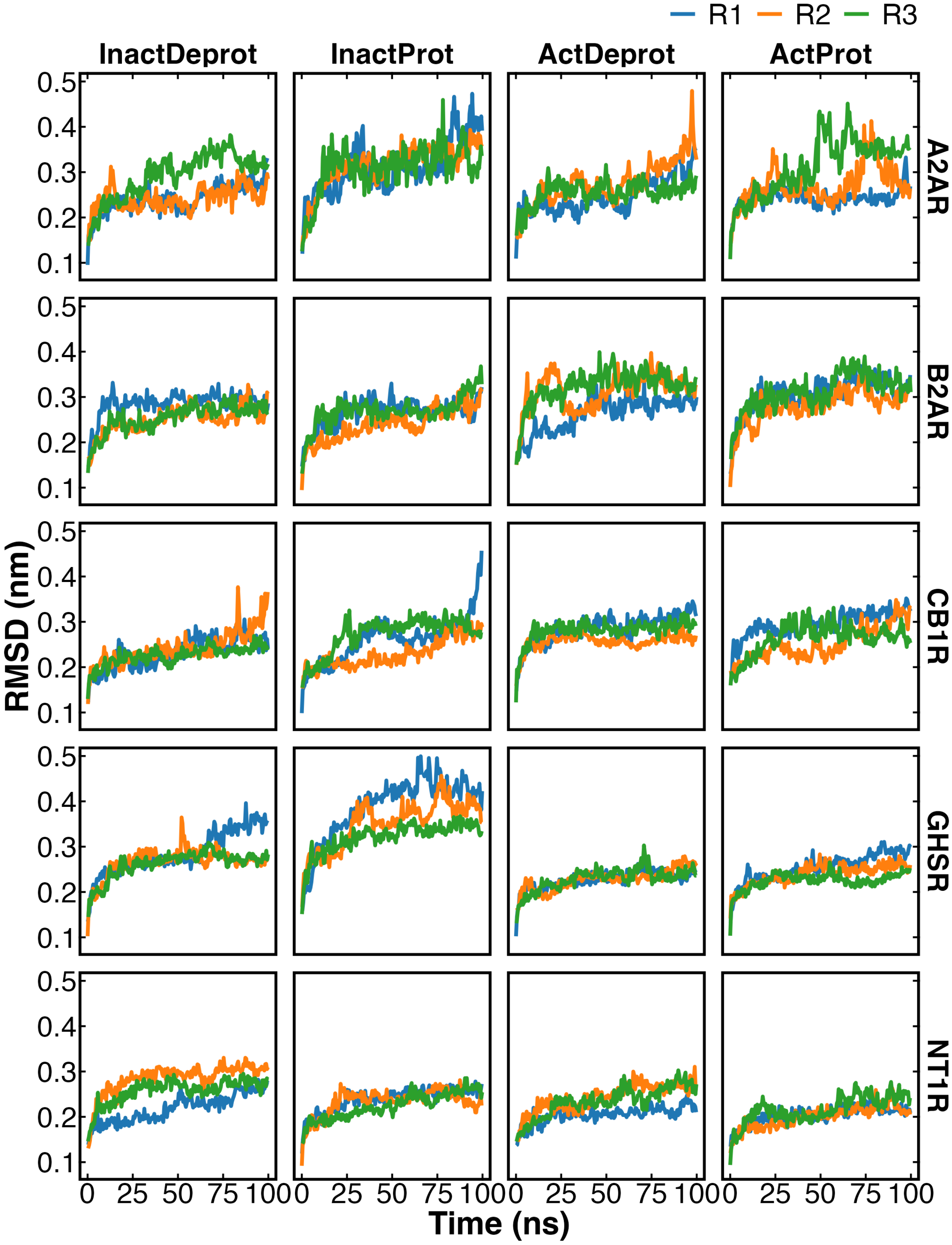

Figure S1: RMSD of TM region throughout the MD simulation time.

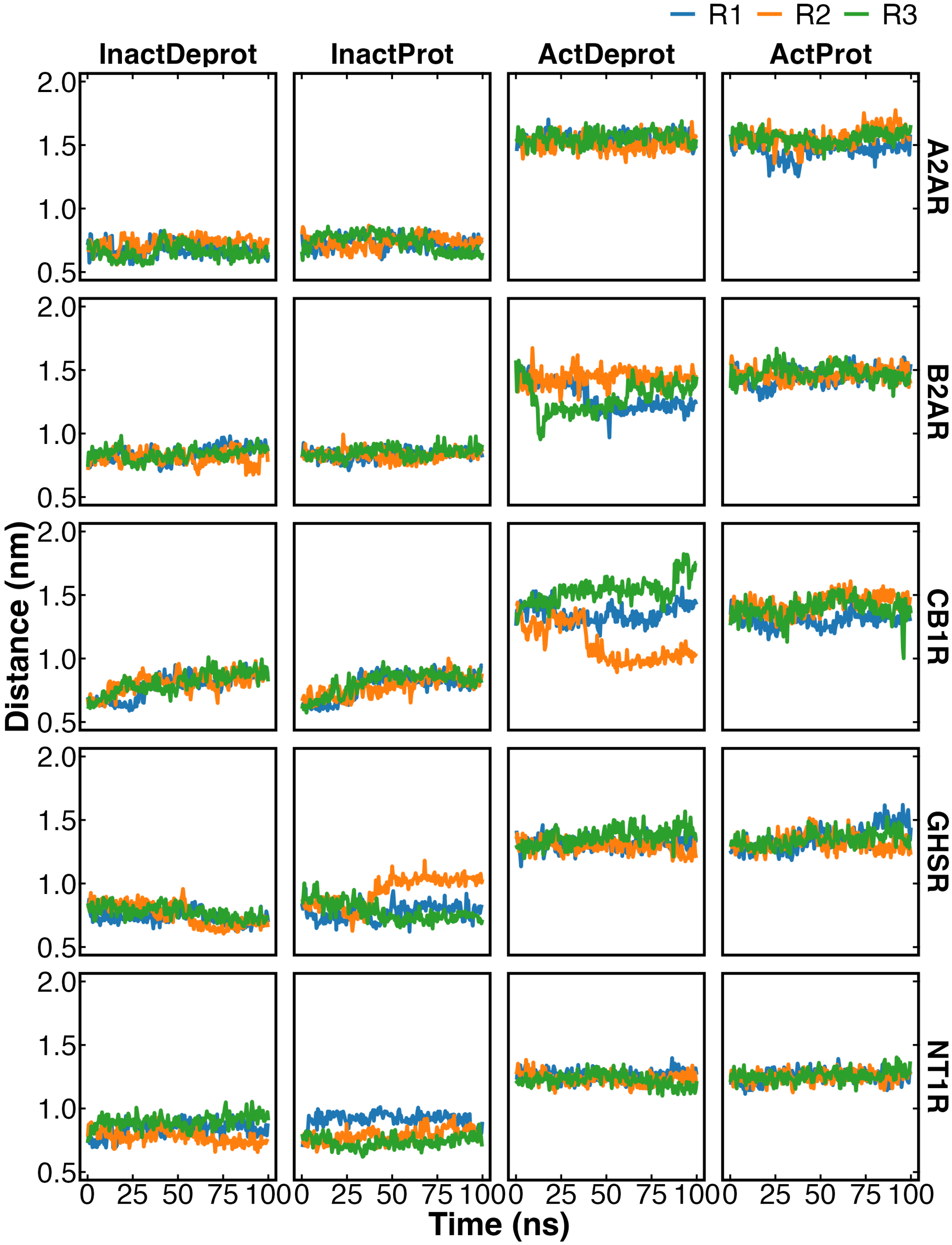

Figure S2: TM3-TM6 distance throughout the MD simulation time.

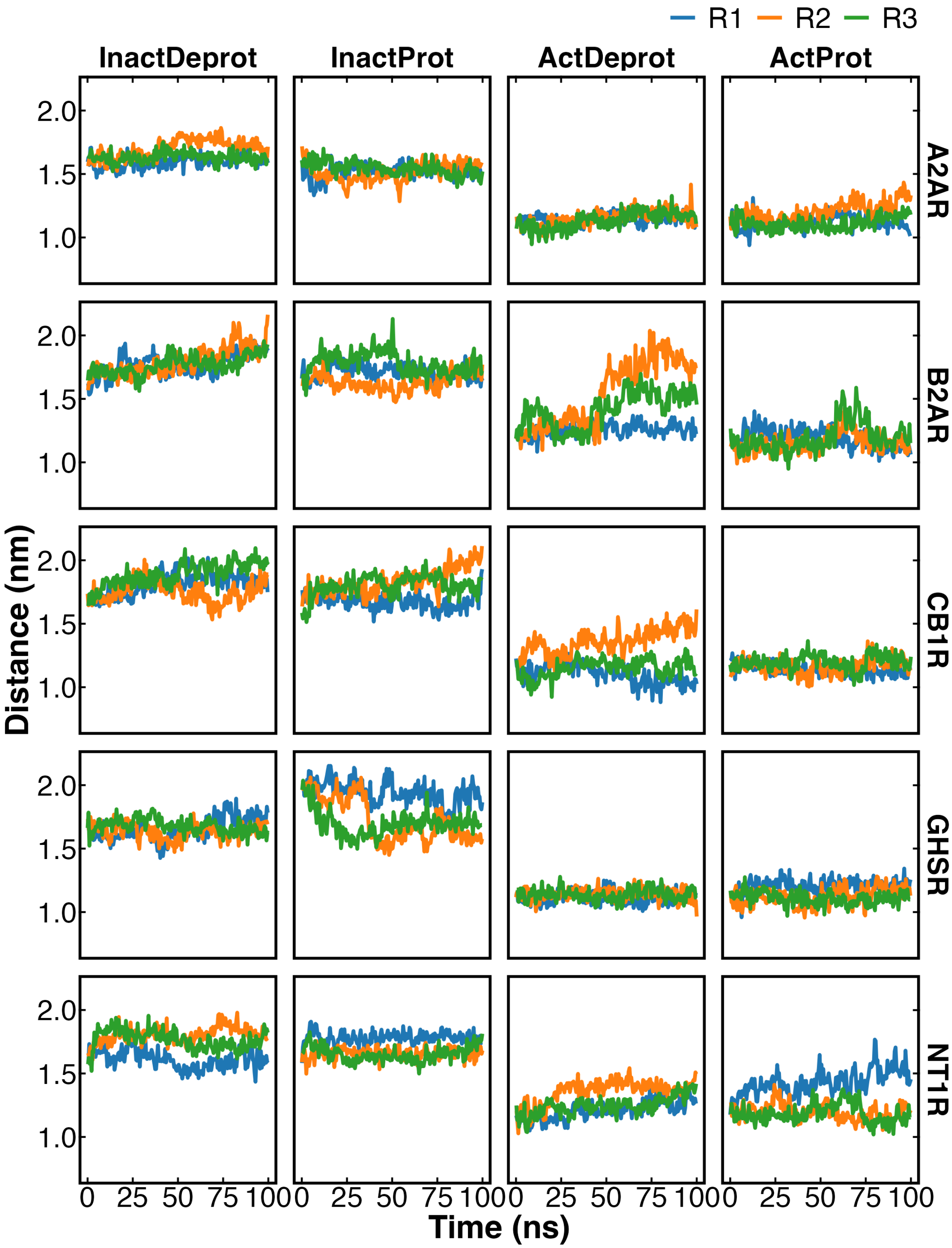

Figure S3: TM3-TM7 distance throughout the MD simulation time.

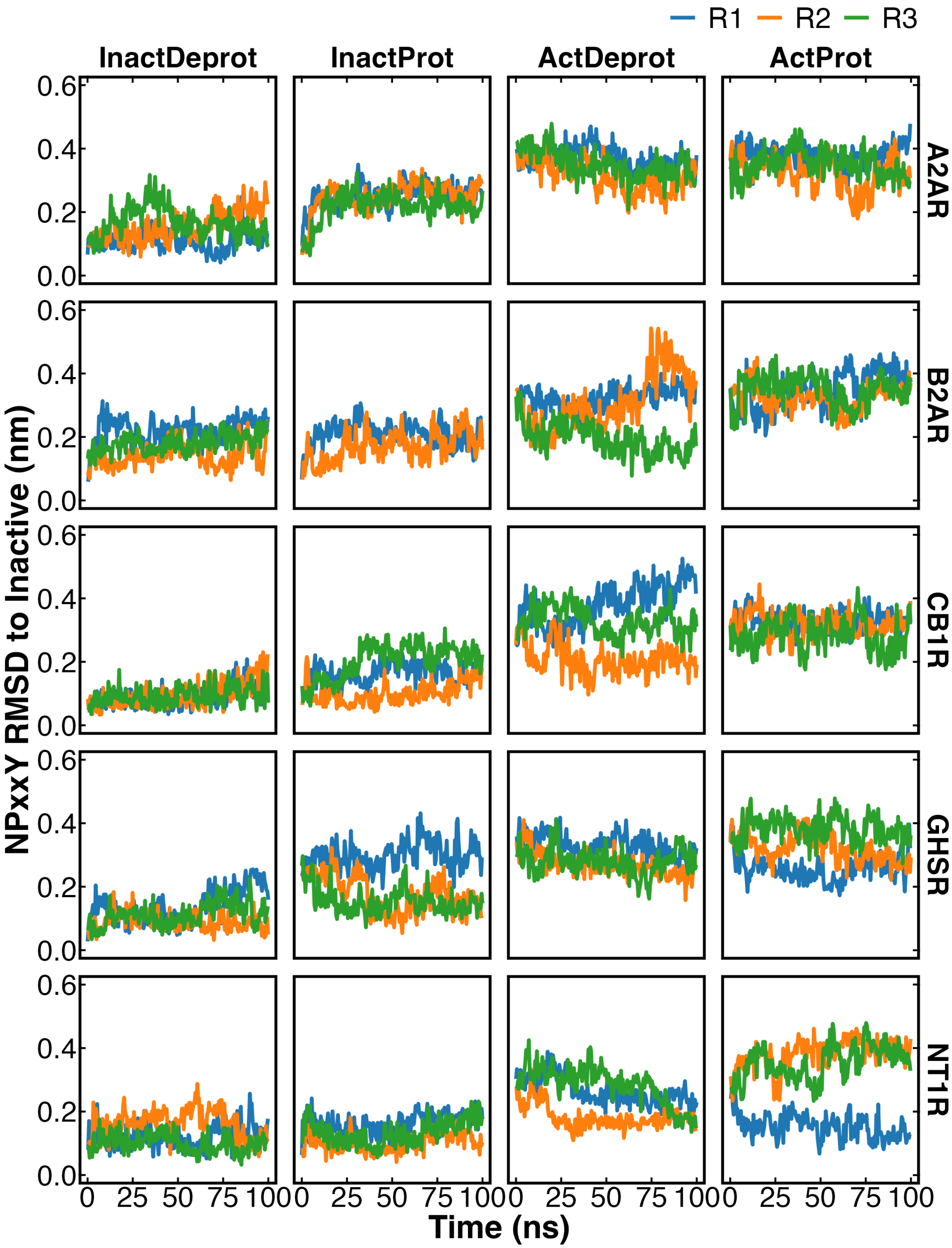

Figure S4: NPxxY RMSD to Inactive throughout the simulation time.

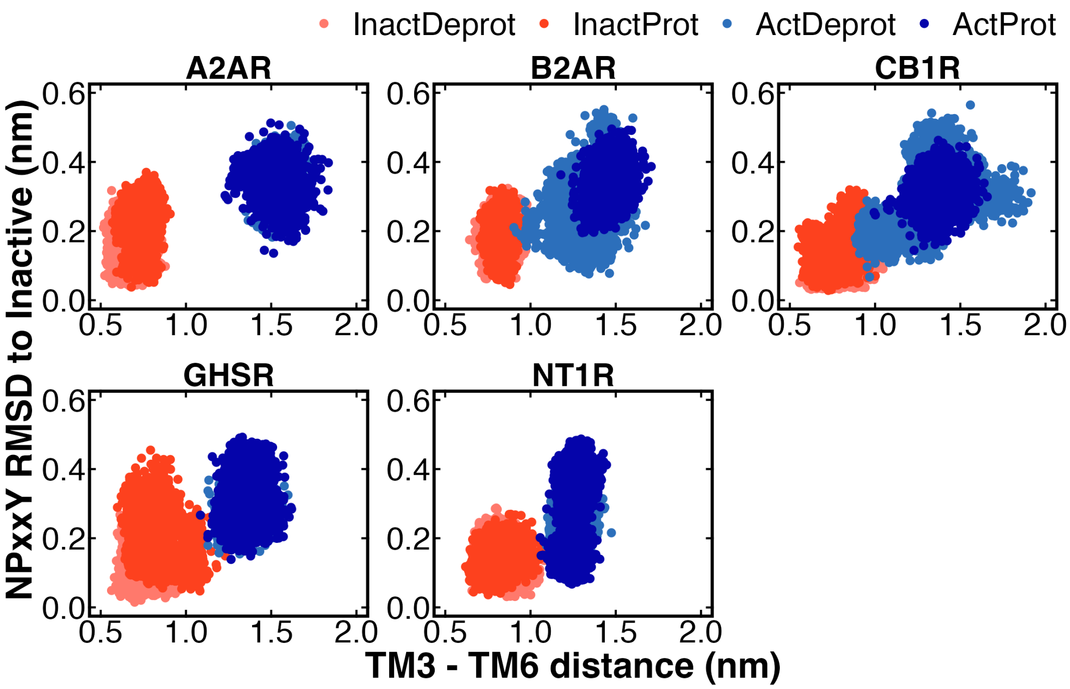

Figure S5: TM3-TM6 distance vs NPxxY RMSD to Inactive.

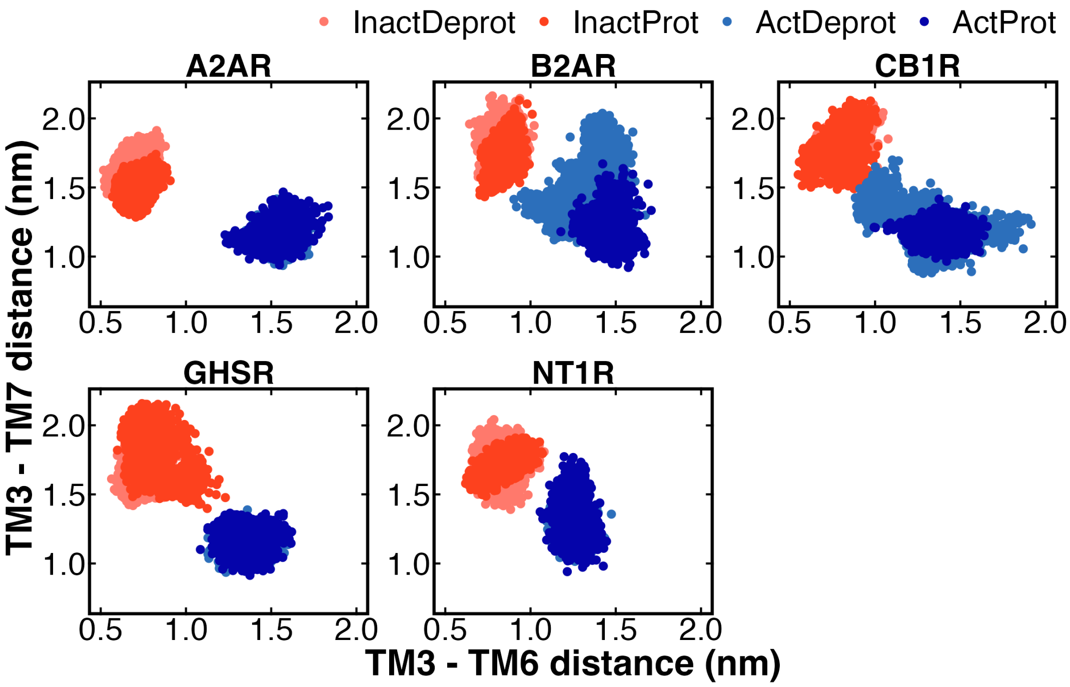

Figure S6: TM3-TM6 distance vs TM3-TM7 distance.

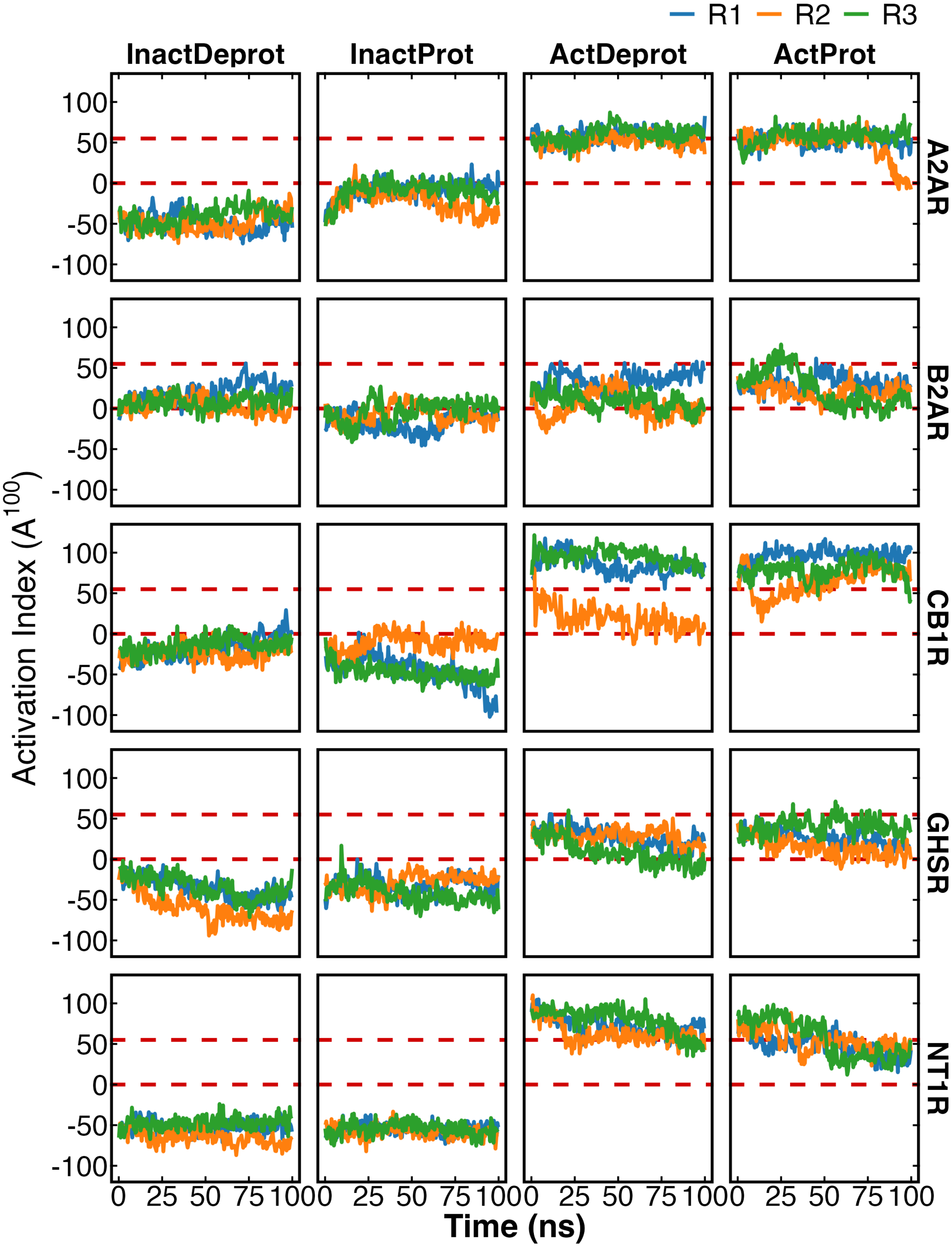

Figure S7: Activation Index throughout the MD simulation time. Red dashed lines mark the limits between Inactive(-50,0), Intermediate(0,55), Active(55,100).

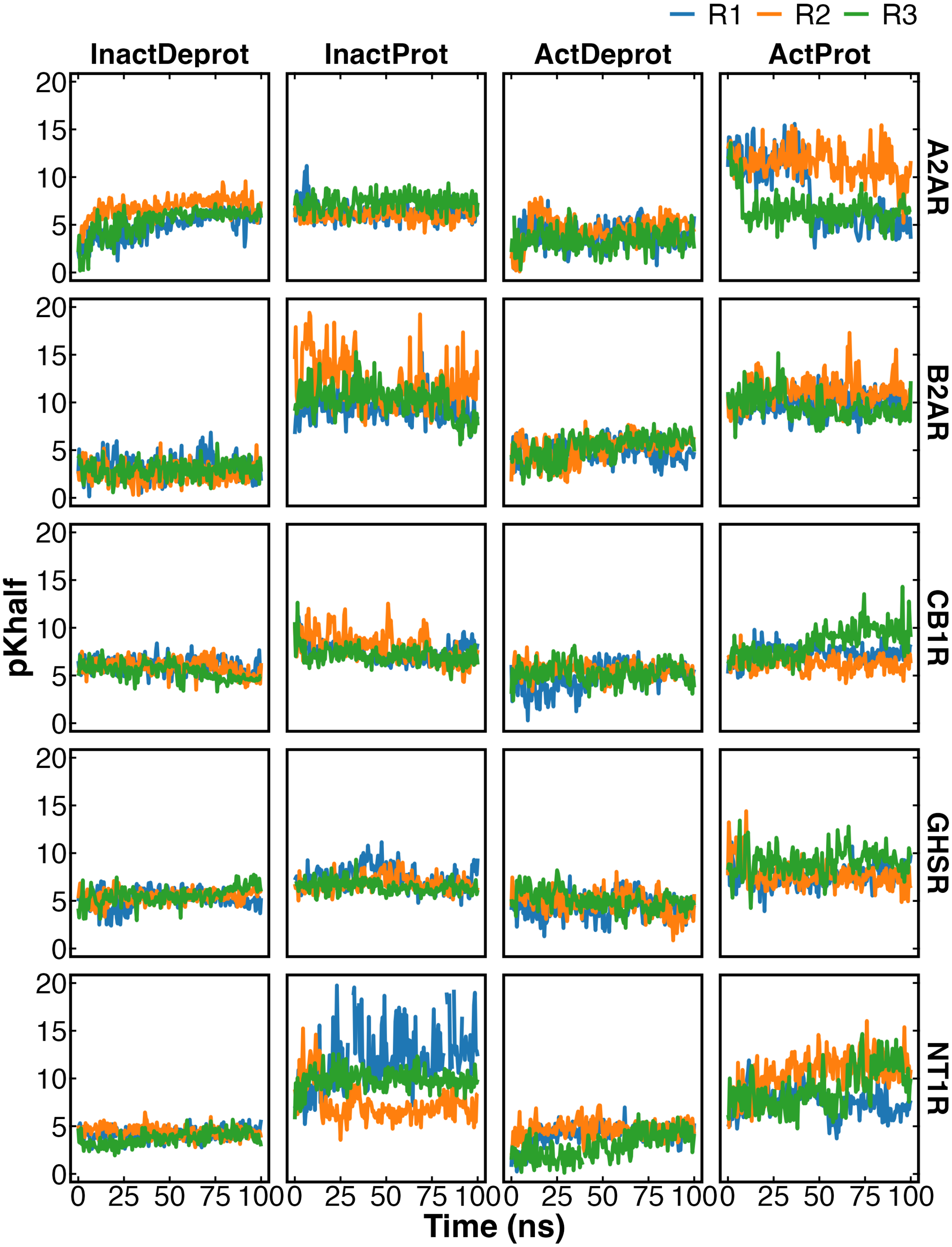

Figure S8: pK_{half} throughout the MD simulation time.

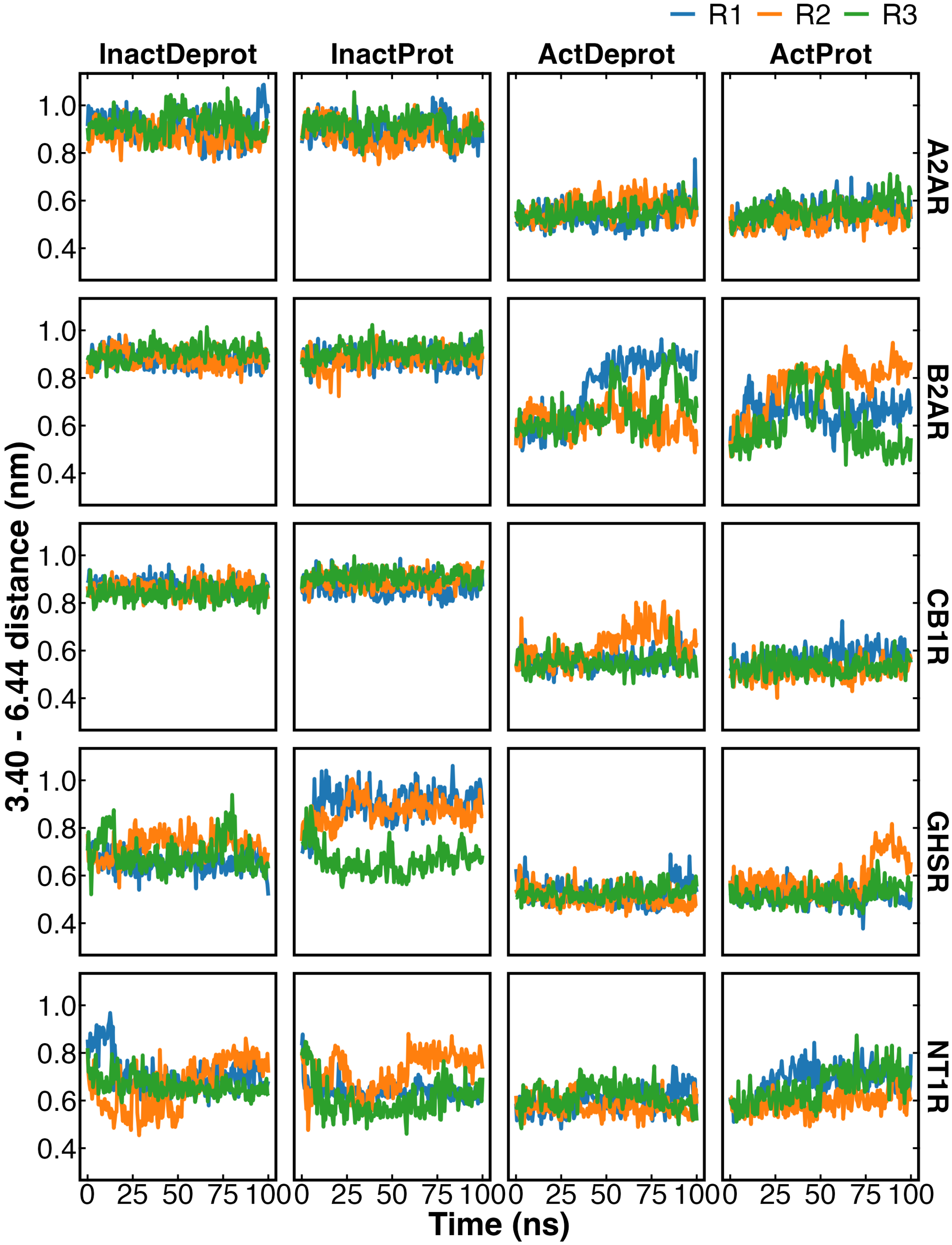

Figure S9: Distance 3.40 - 6.48 throughout the MD simulation time.

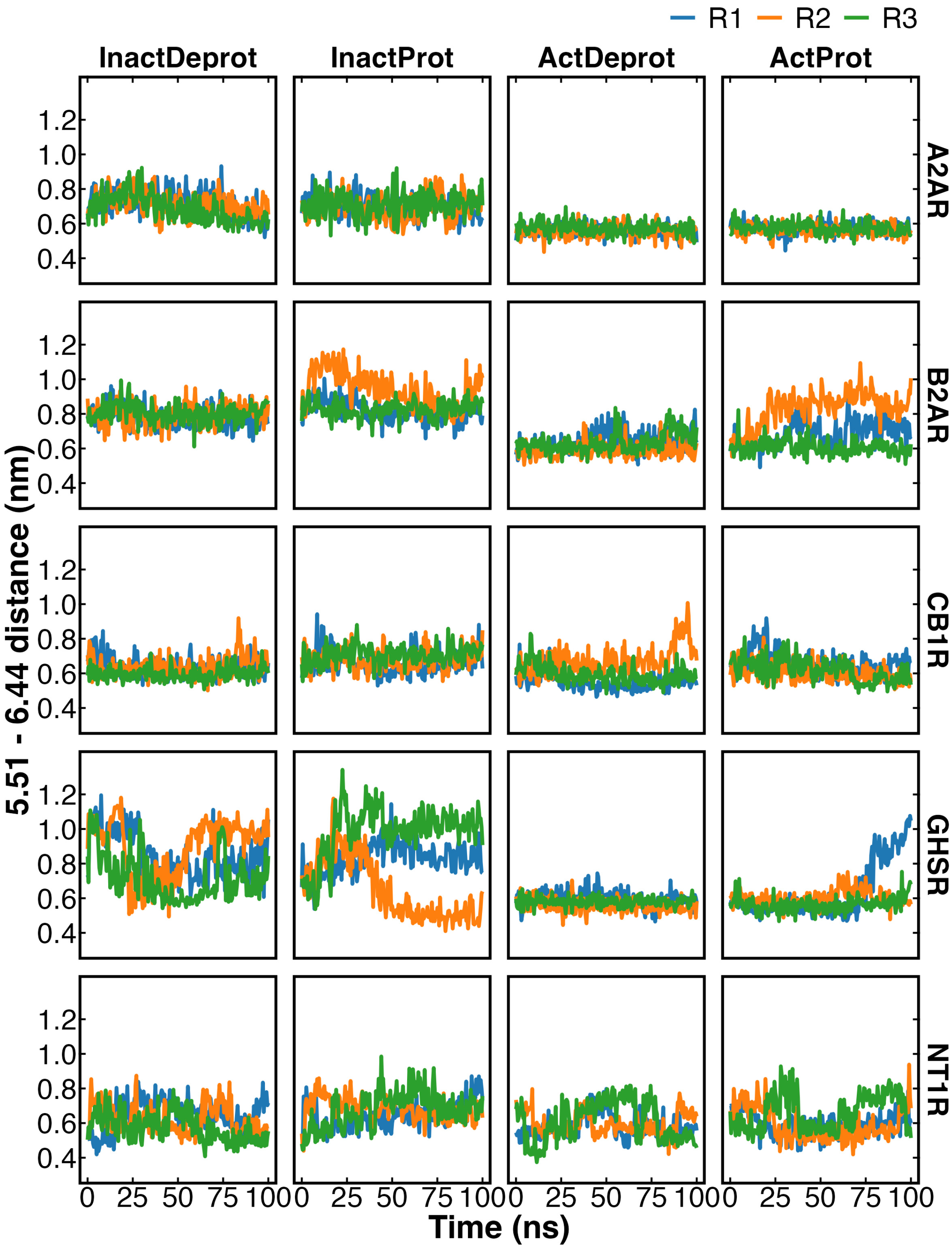

Figure S10: Distance 5.51 - 6.44 throughout the MD simulation time.

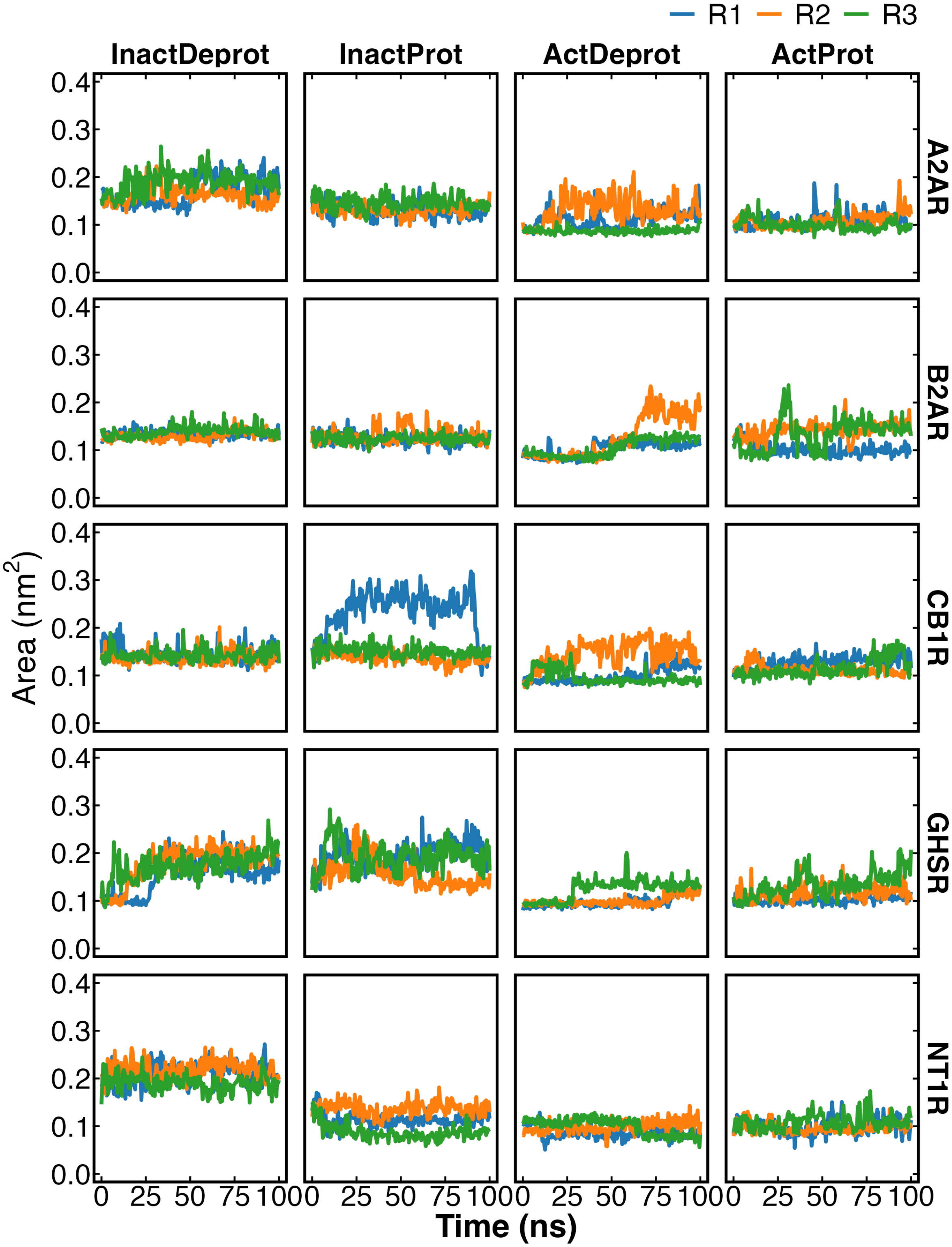

Figure S11: Na^+ pocket area throughout the MD simulation time.

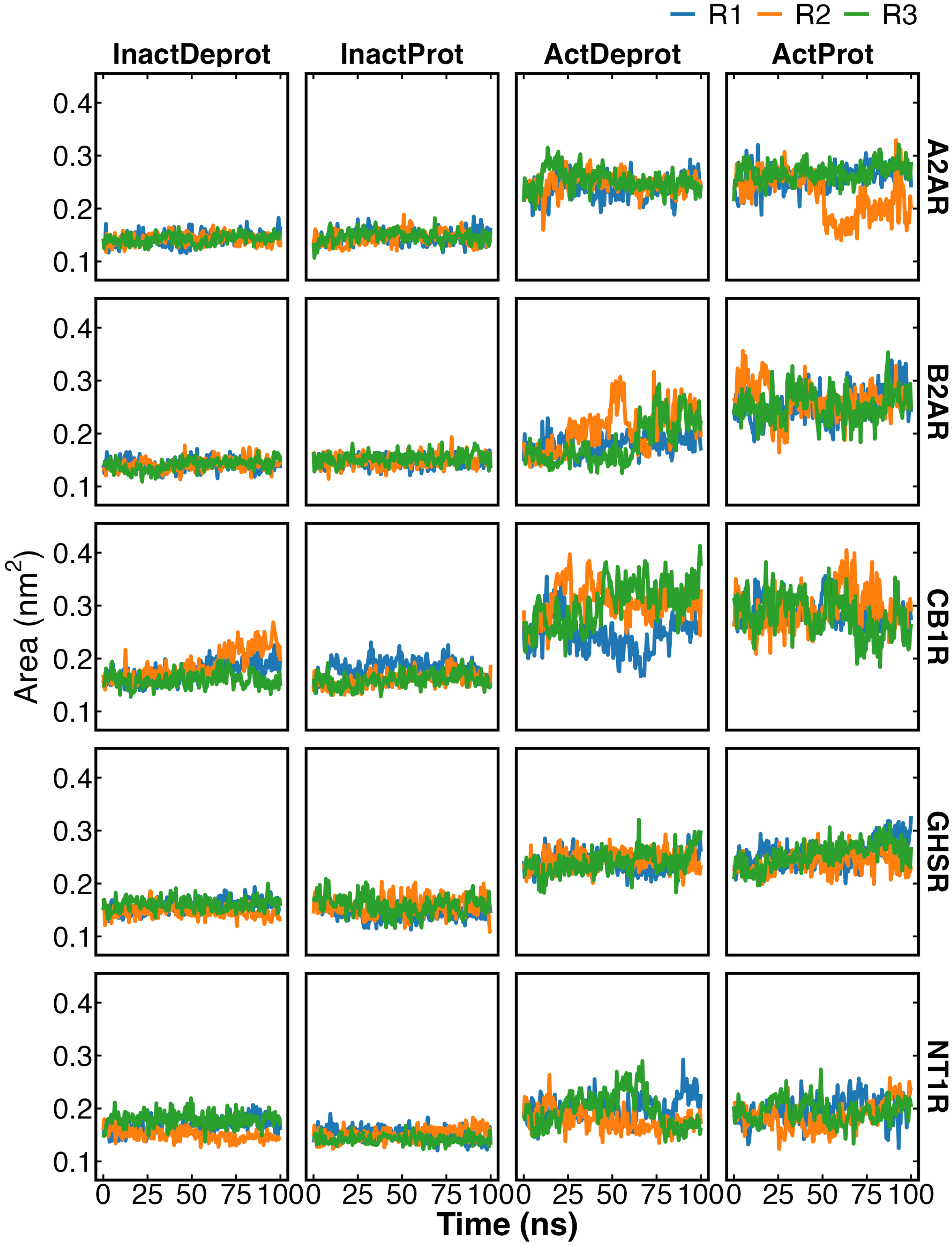

Figure S12: Hydrophobic Lock area throughout the MD simulation time.

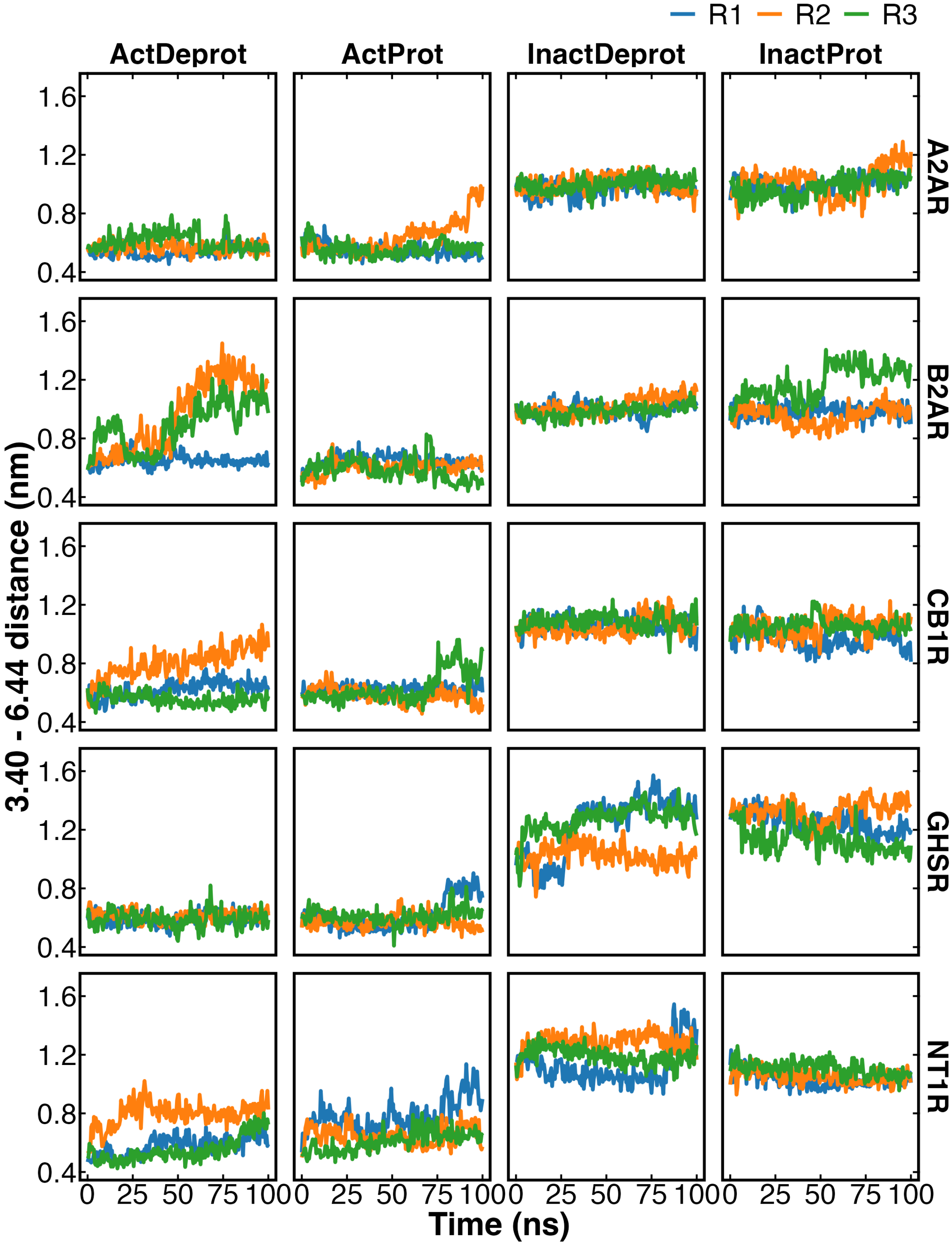

Figure S13: Distance 3.43 - 7.53 throughout the MD simulation time.

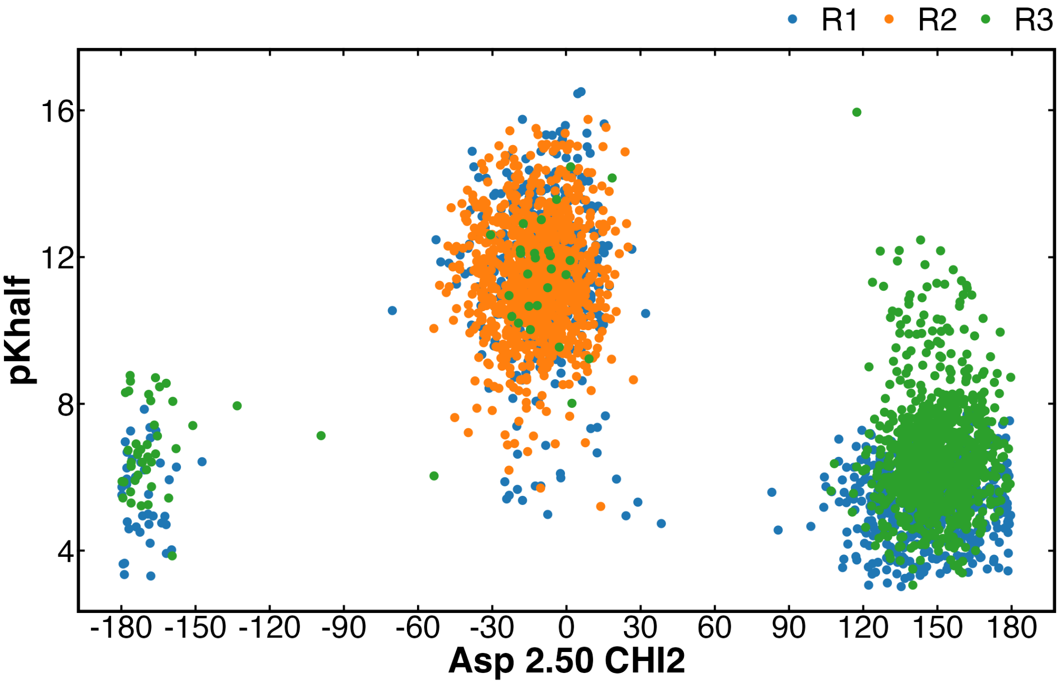

Figure S14: Asp$^{2.50}$ CHI2 flip vs pK_{half} in A2AR^{ActProt} system.

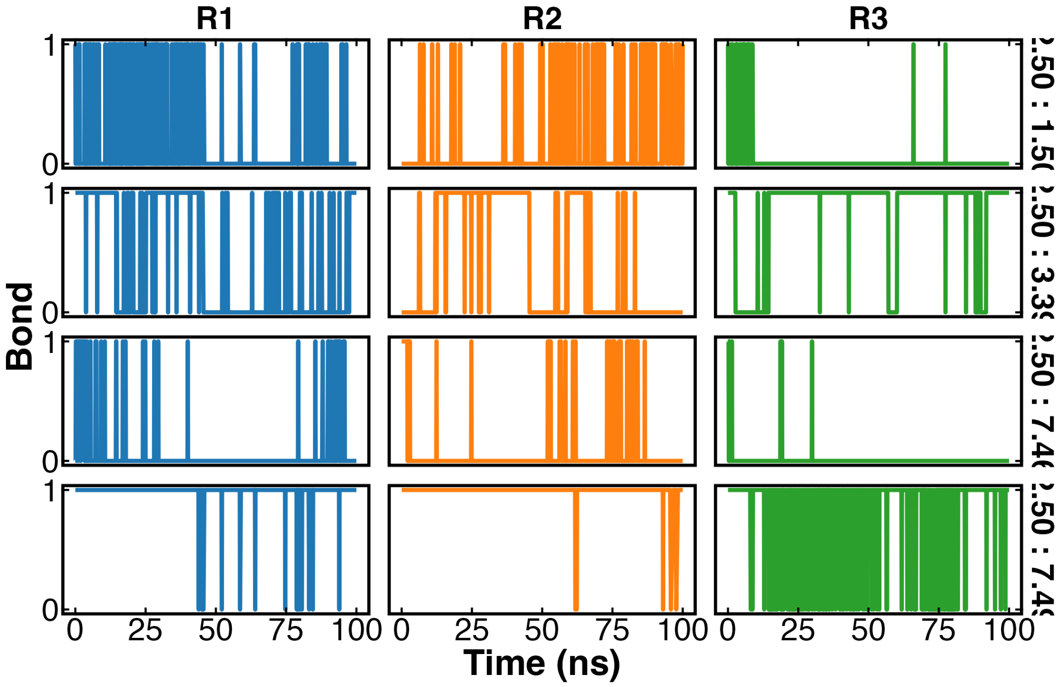

Figure S15: Asp$^{2.50}$ H-bonds throughout time on A2AR^{ActProt} system.

References

(S1) Egloff, P.; Hillenbrand, M.; Klenk, C.; Batyuk, A.; Heine, P.; Balada, S.; Schlinkmann, K. M.; Scott, D. J.; Schütz, M.; Plückthun, A. Structure of signaling-competent neurotensin receptor 1 obtained by directed evolution in *Escherichia Coli*. *Proc. Natl. Acad. Sci. USA* **2014**, *111*, E655–E662.

(S2) Segala, E.; Guo, D.; Cheng, R. K. Y.; Bortolato, A.; Deflorian, F.; Doré, A. S.; Errey, J. C.; Heitman, L. H.; IJzerman, A. P.; Marshall, F. H.; Cooke, R. M. Controlling the Dissociation of Ligands from the Adenosine A_{2A} Receptor through Modulation of Salt Bridge Strength. *J. Med. Chem.* **2016**, *59*, 6470–6479.

(S3) Carpenter, B.; Nehmé, R.; Warne, T.; Leslie, A. G. W.; Tate, C. G. Structure of the adenosine A_{2A} receptor bound to an engineered G protein. *Nature* **2016**, *536*, 104–107.

(S4) Cherezov, V.; Rosenbaum, D. M.; Hanson, M. A.; Rasmussen, S. G. F.; Thian, F. S.; Kobilka, T. S.; Choi, H.-J.; Kuhn, P.; Weis, W. I.; Kobilka, B. K.; Stevens, R. C. High-Resolution Crystal Structure of an Engineered Human β_2-Adrenergic G Protein-Coupled Receptor. *Science* **2007**, *318*, 1258–1265.

(S5) Rasmussen, S. G. F.; DeVree, B. T.; Zou, Y.; Kruse, A. C.; Chung, K. Y.; Kobilka, T. S.; Thian, F. S.; Chae, P. S.; Pardon, E.; Calinski, D.; Mathiesen, J. M.; Shah, S. T. A.; Lyons, J. A.; Caffrey, M.; Gellman, S. H.; Steyaert, J.; Skiniotis, G.; Weis, W. I.; Sunahara, R. K.; Kobilka, B. K. Crystal structure of the β_2 adrenergic receptor–G_s protein complex. *Nature* **2011**, *477*, 549–555.

(S6) Shao, Z.; Yin, J.; Chapman, K.; Grzemska, M.; Clark, L.; Wang, J.; Rosenbaum, D. M. High-resolution crystal structure of the human CB1 cannabinoid receptor. *Nature* **2016**, *540*, 602–606.

(S7) Hua, T.; Li, X.; Wu, L.; Iliopoulos-Tsoutsouvas, C.; Wang, Y.; Wu, M.; Shen, L.; Brust, C. A.; Nikas, S. P.; Song, F.; Song, X.; Yuan, S.; Sun, Q.; Wu, Y.; Jiang, S.;

Grim, T. W.; Benchama, O.; Stahl, E. L.; Zvonok, N.; Zhao, S.; Bohn, L. M.; Makriyannis, A.; Liu, Z.-J. Activation and Signaling Mechanism Revealed by Cannabinoid Receptor-G_i Complex Structures. *Cell* **2020**, *180*, 655–665.e18.

(S8) Shiimura, Y.; Horita, S.; Hamamoto, A.; Asada, H.; Hirata, K.; Tanaka, M.; Mori, K.; Uemura, T.; Kobayashi, T.; Iwata, S.; Kojima, M. Structure of an antagonist-bound ghrelin receptor reveals possible ghrelin recognition mode. *Nat. Commun.* **2020**, *11*, 4160.

(S9) Wang, Y.; Guo, S.; Zhuang, Y.; Yun, Y.; Xu, P.; He, X.; Guo, J.; Yin, W.; Xu, H. E.; Xie, X.; Jiang, Y. Molecular recognition of an acyl-peptide hormone and activation of ghrelin receptor. *Nat. Commun.* **2021**, *12*, 5064.

(S10) Kato, H. E.; Zhang, Y.; Hu, H.; Suomivuori, C.-M.; Kadji, F. M. N.; Aoki, J.; Krishna Kumar, K.; Fonseca, R.; Hilger, D.; Huang, W.; Latorraca, N. R.; Inoue, A.; Dror, R. O.; Kobilka, B. K.; Skiniotis, G. Conformational transitions of a neurotensin receptor 1–G_{i1} Complex. *Nature* **2019**, *572*, 80–85.
